# A Method to Improve Electron Density Measurement of Cone-Beam CT Using Dual Energy Technique

**DOI:** 10.1155/2015/858907

**Published:** 2015-08-05

**Authors:** Kuo Men, Jian-Rong Dai, Ming-Hui Li, Xin-Yuan Chen, Ke Zhang, Yuan Tian, Peng Huang, Ying-Jie Xu

**Affiliations:** Department of Radiation Oncology, Cancer Hospital, Chinese Academy of Medical Sciences and Peking Union Medical College, Beijing 100021, China

## Abstract

*Purpose*. To develop a dual energy imaging method to improve the accuracy of electron density measurement with a cone-beam CT (CBCT) device. *Materials and Methods*. The imaging system is the XVI CBCT system on Elekta Synergy linac. Projection data were acquired with the high and low energy X-ray, respectively, to set up a basis material decomposition model. Virtual phantom simulation and phantoms experiments were carried out for quantitative evaluation of the method. Phantoms were also scanned twice with the high and low energy X-ray, respectively. The data were decomposed into projections of the two basis material coefficients according to the model set up earlier. The two sets of decomposed projections were used to reconstruct CBCT images of the basis material coefficients. Then, the images of electron densities were calculated with these CBCT images. *Results*. The difference between the calculated and theoretical values was within 2% and the correlation coefficient of them was about 1.0. The dual energy imaging method obtained more accurate electron density values and reduced the beam hardening artifacts obviously. *Conclusion*. A novel dual energy CBCT imaging method to calculate the electron densities was developed. It can acquire more accurate values and provide a platform potentially for dose calculation.

## 1. Introduction

Radiotherapy aims to deliver sufficient dose to the tumor target and spare the organ at risk (OAR) around target to achieve the goal, that is, killing the tumor cell with minimum toxicity to the normal tissues. Advanced irradiation techniques such as intensity modulated radiotherapy (IMRT) [1], volumetric modulated arc therapy (VMAT) [2], and stereotactic radiation therapy (SRT) [3] can generate complex dose distribution with high dose areas firmly conformed to the target. Because of the high dose gradients at the boundary of target, the anatomical change due to weight loss, tumor shrinkage, and growth during the treatment will lead to inaccurate dose delivered to patient with respect to the initial planning. To address this problem, imaging is quite necessary in procedures of radiotherapy treatment specifically in the image guided radiation therapy (IGRT) and the adaptive radiation therapy (ART).

Cone-beam computed tomography (CBCT), implemented on linear accelerators, has been widely used in the clinic as the mainstream technology of IGRT [4–6]. It can provide volumetric information of a patient at the treatment position. Therefore it is used to verify the patient setup and detect the change of the tumor location and then correct it if necessary before treatment. Moreover, the CBCT images have the potential to delineate the patient anatomy online and determine the tissues' electron densities which can be used to calculate the dose for adaptive treatment planning. The conversion of the CT number (in Hounsfield units, HU) into an electron density (*ρ*_*e*_) enables the treatment planning system (TPS) to account for tissue heterogeneities. Therefore, the accuracy of patient dose calculations is largely dependent on the accuracy of the electron density.

One limitation of CBCT image quality is the serious artifacts introduced by X-ray scatter [7] and beam hardening [8]. This will reduce contrast resolution and increase noise in CBCT images, therefore affecting the HU and electron density values.

On the other hand, the HU-*ρ*_*e*_ conversion is usually performed using a calibration phantom containing several modules with known electron densities. However, the CT numbers depend on not only the electron densities but also the effective atomic numbers. And the elemental composition of the modules is different from that of human tissues. As a result, *ρ*_*e*_ of tissues cannot be exactly converted from the CT numbers via a voxel-to-voxel correspondence and the HU-*ρ*_*e*_ conversion curve is often divided into several parts, assuming a linear relationship in each part [9].

Dual energy CT, first proposed by Alvarez and MacOvski [10], is a significant technological advancement in imaging. By obtaining CT data at high and low X-ray energies, dual energy CT can provide both electron density (*ρ*_*e*_) and effective atomic number (*Z*_eff_), thus facilitating tissue type identification. It has been a hot topic in both research and application in the diagnosis field [11–16]. However, the advantages of dual energy CBCT in radiotherapy need to be further researched.

The overall purpose of this paper is to implement a dual energy CBCT imaging method to improve the *ρ*_*e*_ measurement by reducing the beam hardening and determining the electron densities of human tissues directly without using the problematic HU-*ρ*_*e*_ conversion curve. The method uses a CBCT imaging device mounted on a linear accelerator and two scans are performed to acquire high and low kVp images. The performance of the proposed method was evaluated using a virtual phantom simulation and experiments of a CTP 486 module and a CIRS model.

## 2. Materials and Methods

### 2.1. Imaging System

This work was performed using the X-ray volumetric imaging (XVI) CBCT system of an Elekta Synergy machine. The X-ray source uses a rotating anode X-ray tube (Dunlee D604, Aurora, IL) with peak tube potential of 150 kVp and maximum current of 500 mA. The kV source arm contains two slots for fitting a collimator cassette and a filtration cassette. The detector is an indirect detection flat-panel imager with a spatial resolution of 1,024 × 1,024 array of 0.4 × 0.4 mm^2^ pixels (RID1640-A11, Perkin Elmer, Wiesbaden, Germany). The source-to-axis distance and source-to-detector distance are 1,000 and 1,536 mm, respectively. Projection images can be acquired with three different “fields of view” (FOVs): small (S), medium (M), and large (L). The number of projections for a full 360-degree rotation is approximately 600. The XVI software uses a cone-beam reconstruction process based on the Feldkamp-Davis-Kress (FDK) algorithm.

### 2.2. Basic Principles of Dual Energy Imaging Method

Dual energy imaging method is fundamentally derived from the fact that the X-ray absorption linear attenuation coefficient (*μ*) of an element depends on photon energy (*E*). In the diagnostic energy range, the attenuation coefficient of a material (*μ*(*E*)) can be approximated by a linear combination of two basis materials [4, 12]:

(1) $$\begin{array}{c} \mu \left(\;E\;\right)=b_{1}\mu_{1}\left(\;E\;\right)+b_{2}\mu_{2}\left(\;E\;\right), \end{array}$$

where *μ*_1_(*E*) and *μ*_2_(*E*) are the attenuation coefficient of the two basis materials and *b*_1_ and *b*_2_ are named as the basis material coefficients, respectively.

In dual energy imaging, the phantom is scanned twice with the peak tube potential of 120 kVp (high energy) and 70 kVp (low energy), respectively. For the high and low energy X-ray spectra *S*_*H*_(*E*) and *S*_*L*_(*E*), the measured projections *g*_*H*_ and *g*_*L*_ are [4]

(2) gHln⁡IHI0=ln⁡∫0EmHSHEexp⁡−B1μ1E−B2μ2EdE,gLln⁡ILI0=ln⁡∫0EmLSLEexp⁡−B1μ1E−B2μ2EdE,

where *B*_*i*_ is the projection of basis material coefficient *b*_*i*_ and is represented by

(3) $$\begin{array}{c} B_{i}=\overset{}{\underset{l}{\int }}b_{i}\left(\;x,y\;\right)dl,\;\left(i=1,2\right). \end{array}$$

The core of dual energy imaging process is to calculate the basis material coefficient *b*_*i*_ that can be derived from the attenuation measured at two different energy spectra. Once *b*_*i*_ are determined, the electron density (*ρ*_*e*_) can be calculated by [13]

(4) $$\begin{array}{c} \rho_{e}=b_{1}\rho_{e1}+b_{2}\rho_{e2}, \end{array}$$

where *ρ*_*e*1_ and *ρ*_*e*2_ are the electron densities of the two basis materials, respectively.

### 2.3. Basis Material Coefficients Calculation

A key step of this method is to calculate the basis material coefficients calculation (*b*_1_ and *b*_2_) from the dual energy gray projections (*g*_*H*_ and *g*_*L*_).

When the X-ray passes through the two basis materials with path lengths of *d*_1_ and *d*_2_, the projections *g*_*H*_ and *g*_*L*_ measured with high and low energy X-ray spectra *S*_*H*_(*E*) and *S*_*L*_(*E*) are

(5) gHln⁡IHI0=ln⁡∫0EmHSHEexp⁡−d1μ1E−d2μ2EdE,gLln⁡ILI0=ln⁡∫0EmLSLEexp⁡−d1μ1E−d2μ2EdE.

As can be seen from (2) and (5), if the measurements (*g*_*H*_, *g*_*L*_) are the same in the two formulas, the pair (*B*_1_, *B*_2_) equals the X-ray path length pair (*d*_1_, *d*_2_) numerically. Because *B*_*i*_ is the projection of *b*_*i*_, the basis material coefficient *b*_*i*_ can be reconstructed from *B*_*i*_ using the FDK algorithm.

In our method, projections (*g*_*H*_, *g*_*L*_) of the basis materials were acquired by changing the X-ray path lengths (*d*_1_, *d*_2_) through the two basis materials. Once a set of projections that covered the desired range were obtained for the respective lengths of basis material, a look-up table was generated. The look-up table related a pair of projection values (e.g., *g*_*H*_ and *g*_*L*_) at high and low energy spectra to a pair of path lengths of basis materials (e.g., *d*_1_ and *d*_2_). After the object was scanned, this look-up table was used to convert the dual energy gray projections (*g*_*H*_, *g*_*L*_) to the path lengths (*d*_1_, *d*_2_). Then the 2D projections of path lengths (*d*_1_, *d*_2_), which equaled (*B*_1_, *B*_2_), were used to reconstruct a 3D image of the basis material coefficient (*b*_1_, *b*_2_).

In the process of creating the look-up table, the projection data (*g*_*H*_, *g*_*L*_) of the basis materials were also affected by the beam hardening effect. As a result, when the look-up table was used to calculate *d*_1_ and *d*_2_, the beam hardening effect had already been taken into account and this method was expected to reduce the beam hardening artifact.

### 2.4. The Process of Dual Energy Imaging

**Figure 1:**
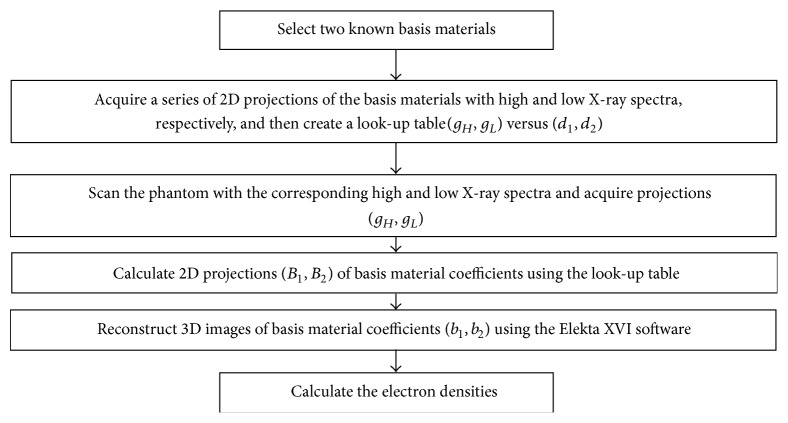
The flowchart of the proposed method.

The flowchart in Figure 1 shows the implementation procedure of the proposed method. The procedure had six steps: (1) select two known basis materials; (2) acquire a series of 2D projections of the basis materials with high and low X-ray spectra, respectively, and then create a look-up table (*g*_*H*_, *g*_*L*_) versus (*d*_1_, *d*_2_); (3) scan the phantom with the corresponding high and low X-ray spectra and acquire projections (*g*_*H*_, *g*_*L*_); (4) calculate the 2D projections (*B*_1_, *B*_2_) of basis material coefficients using the look-up table; (5) reconstruct the 3D images of basis material coefficients (*b*_1_, *b*_2_) using the Elekta XVI software; (6) calculate the electron densities according to (4).

### 2.5. Basis Material

**Figure 2:**
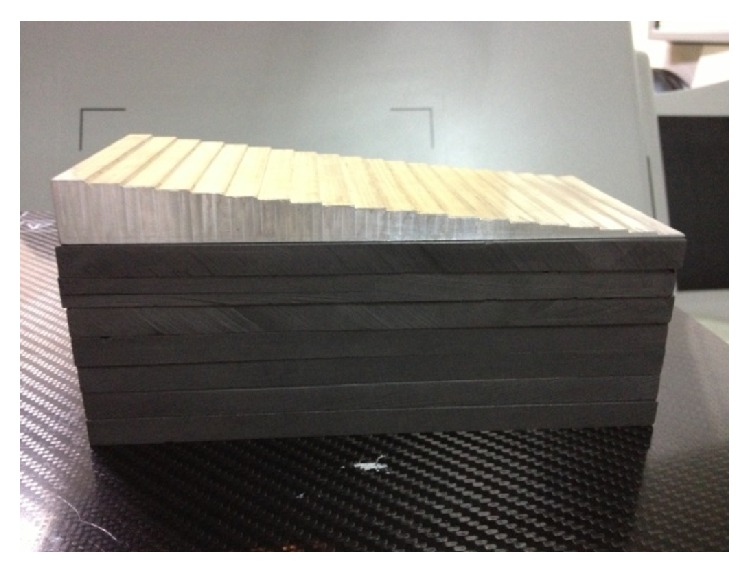
Basis material phantom.

The basis materials used for imaging in this study were graphite (C) and aluminum (Al) whose atomic number was 6 and 13, respectively. The tissue-like material graphite was made in the shape of cuboid, while the bone-like material aluminum was made in the shape of step-wedge (Figure 2). The thickness of graphite was 10 mm per slice. The overall slices of graphite were 16. The aluminum step-wedge was ranked from 1 mm to 20 mm, in steps of 1 mm.

### 2.6. Performance Evaluation

#### 2.6.1. Virtual Phantom Simulation

**Figure 3:**
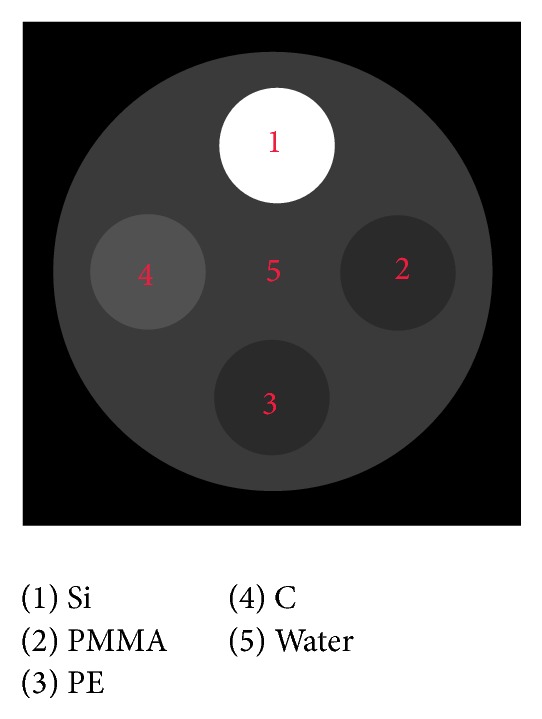
The transaxial arrangement of the virtual phantom.

The above method was first evaluated with a virtual phantom. The transaxial arrangement of the virtual phantom in our simulation was shown in Figure 3. It was a cylinder of diameter 256 mm made of water, with four cylindrical inserts made of silicon (Si), polymethyl methacrylate (PMMA), polyethylene (PE), and graphite (C), respectively. The physical parameters of all the materials were obtained from the NIST database. In the simulation, 512 × 512 detector pixels with 1.0 × 1.0 mm^2^ resolution were used. The source-to-axis distance and source-to-detector distance were 1,000 and 1,536 mm, respectively. The virtual phantom was assumed to be at the center of the imaging FOV. The simulation imitated the actual imaging process of XVI. The CBCT scanner spectra were generated with SpectrumGUI [17]. The peak tube potential of high and low energy spectra was 120 kVp and 70 kVp, respectively. The projections were acquired every 0.6 degrees and the overall number of projections was about 600.

#### 2.6.2. Phantoms Experiments

**Figure 4:**
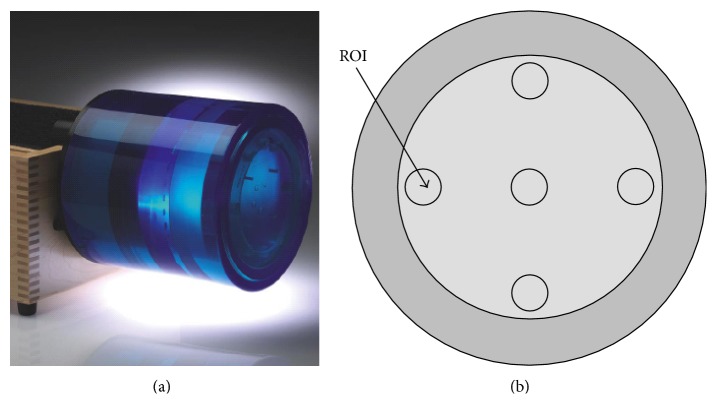
The CTP 486 module within Catphan 500 phantom. (a) Catphan 500 phantom. (b) Schematic drawing of CTP 486 module.

**Figure 5:**
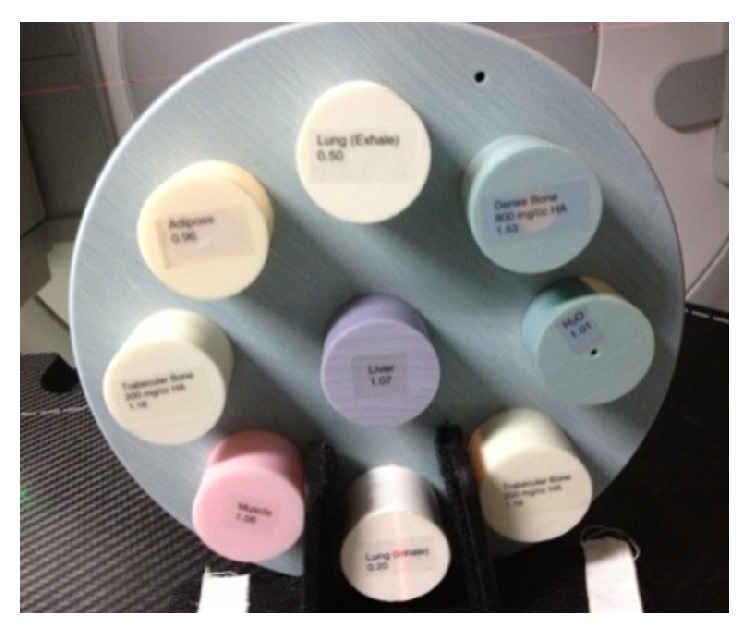
The CIRS phantom.

The method was then evaluated with the CTP 486 module in the Catphan 500 phantom (Figure 4, The Phantom Laboratory, Salem, NY) and an electron density phantom of the CIRS model 062 (Figure 5, Computerized Imaging Reference Systems Inc., Norfolk, VA).

The CTP 486 module was uniform and water-equivalent, with CT number (HU) within 2% (20 HU) of water's density at standard scanning protocols. It had a diameter of 150 mm and high radial and axial uniformity, therefore, an ideal substitute for water. Five regions of interest (ROIs) of size 5 × 5 mm^2^ (about 10 × 10 voxels), including one at the center and four at peripheral positions, were selected optionally to measure the mean in HU values. Beam hardening (BH) was evaluated as the maximum relative difference between the HU of central ROI and the mean HU of the four peripheral ROIs (6). Smaller BH value represented less beam hardening effect:

(6) $$\begin{array}{c} \text{BH}=\left(\;\frac{{\text{Mean}}_{\text{periphery}}-{\text{Mean}}_{\text{center}}}{{\text{Mean}}_{\text{center}}}\;\right)\times 100. \end{array}$$

The CIRS phantom was a cylinder of diameter 180 mm. Nine holes, including one at the center and eight at peripheral positions in the form of concentric circles, were arranged to accommodate a series of the inserts with known electron densities. ROIs of size 5 × 5 mm^2^ (about 10 × 10 voxels) within each insert were used to measure the mean value of the electron densities. The results obtained by the conventional method and dual energy imaging method were compared with the theoretical values.

The phantoms were positioned at the center of the imaging FOV with the aid of room lasers. Images of the phantom were acquired using high and low energy X-ray spectra. The collimator S10 and the filter F0 were used in this study. S10 collimator generated 10 cm small field of view at the isocenter. F0 filter is blank and has no effect on the X-ray beam. For the high energy spectra, the imaging parameters were the tube peak potential of 120 kVp, the tube current of 20 mA, and the exposure time of 20 ms per projection. For the low energy spectra, the imaging parameters were the peak tube potential of 70 kVp, the tube current of 40 mA, and the exposure time of 40 ms per projection. The projection images were processed at a high resolution to yield projections of dimension 1,024 × 1,024 with pixel size of 0.4 × 0.4 mm^2^. The CBCT images were reconstructed at the high resolution with voxel size of 0.5 × 0.5 × 0.5 mm^3^.

## 3. Results

### 3.1. Virtual Phantom Simulation

Table 1 lists the calculated and theoretical values of *ρ*_*e*_. The theoretical values were adopted from the NIST database. The error was less than 1.0% for the calculated values and the correlation coefficient between them was about 1.0.

### 3.2. Phantoms Experiments

**Figure 6:**
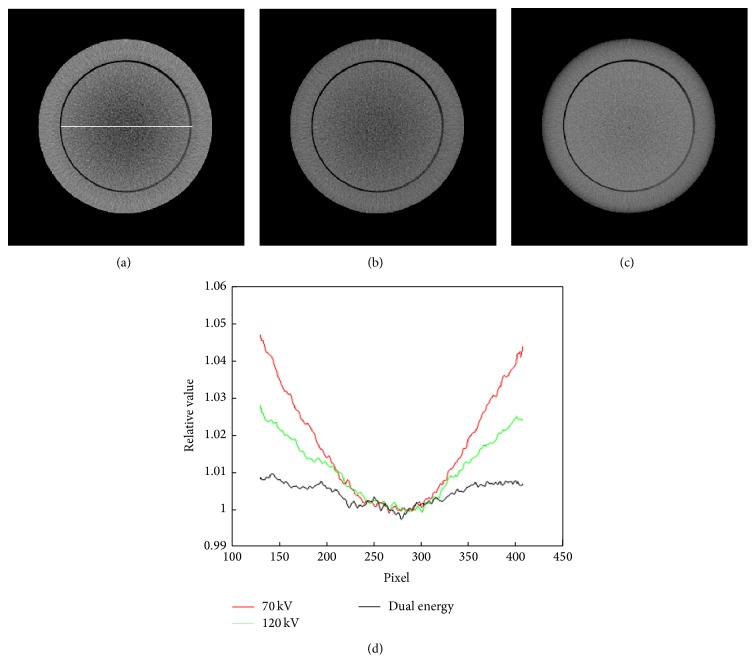
CBCT images of the CTP 486 module in Catphan 500 phantom: (a) 70 kVp, (b) 120 kVp, and (c) dual energy imaging. (d) Profiles through the region of interest, indicated by the white solid line in (a).

Figures 6(a), 6(b), and 6(c) show CBCT images of the CTP 486 module in Catphan 500 phantom acquired with the tube peak potential of 70 kVp, 120 kVp, and dual energy imaging, respectively. The images had the same window level and window width. The values of BH were 4.73, 2.85, and 0.96, respectively.  Figure 6(d) shows profiles through the images, indicated by the white solid line. After the implementation of dual energy imaging, the beam hardening artifact, a cup-like shape (higher at the edges going down towards the center), was reduced obviously.

**Figure 7:**
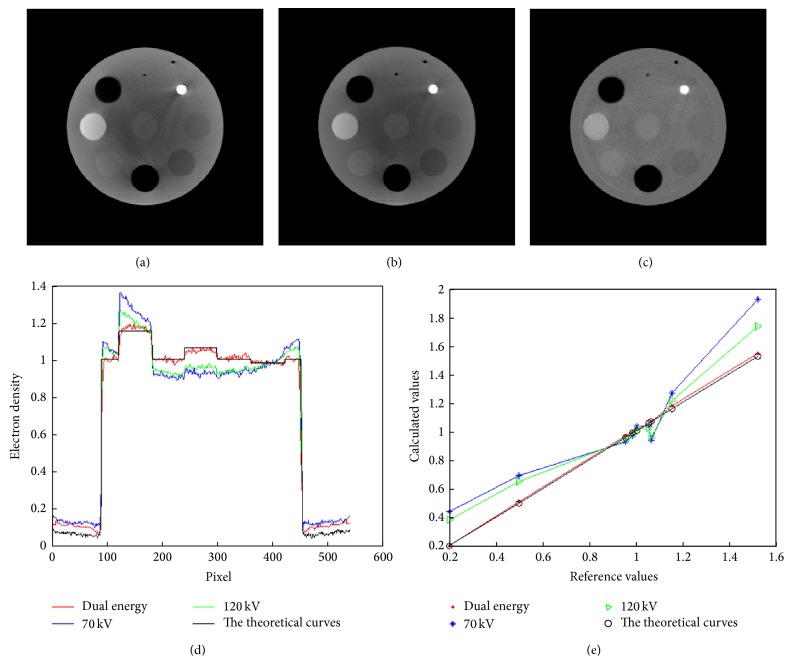
CBCT images of the CIRS phantom: (a) 70 kVp, (b) 120 kVp, and (c) dual energy imaging. (d) Profiles through the horizontal axis of (a), (b), and (c) and the theoretical curves. (e) Electron densities obtained by conventional and dual energy method.

Figure 7 shows the experimental results on the CIRS phantom.  Figure 7(d) shows the phantom's electron density curves determined by conventional CBCT and dual energy imaging method.  Figure 7(e) shows the profiles through the horizontal axis of Figures 7(a), 7(b), and 7(c) and the theoretical curves. Compared with the theoretical values of the phantom, the dual energy imaging method can obtain more accurate electron density values than the conventional CBCT, and the difference with the theoretical values was within 2%. The beam hardening artifact nearly disappeared, as it should be.

Using a PC with an Intel Pentium processor at 3.0 GHz, the additional computation time for a pair of dual energy projection images of dimension 1024 × 1024 was approximately 20 ms and therefore about 12 s to correct all projection images (about 600 frames) of one scan.

## 4. Discussion and Conclusion

As shown in the results, dual energy imaging method can reduce the beam hardening artifacts greatly. This can be explained as follows: the beam hardening artifact is mainly caused by beam hardening which is seen with polychromatic X-ray sources. As the X-ray passes through the body, low energy X-ray photons are attenuated more easily, and the remaining high energy photons are not attenuated as easily. Thus, beam transmission does not follow the simple exponential decay seen with a monochromatic X-ray. In the process of creating the look-up table we acquired projections (*g*_*H*_, *g*_*L*_) of basis materials by varying the X-ray path lengths (*d*_1_, *d*_2_) through the two basis materials. The projection data (*g*_*H*_, *g*_*L*_) were also affected by the beam hardening effect. So the look-up table had already taken the beam hardening effect into account.

CBCT could provide the volumetric data of target and surrounding anatomical structures of patient. However, the image quality of CBCT is usually not good enough for the adaptive radiotherapy, which needs accurate electron density values for dose calculation. This study shows that the dual energy method can obtain more accurate electron densities distribution of patient at the treatment position. The acquisition of dual energy CBCT image would cost only about 120 seconds just before the delivery of each treatment fraction. Then, if necessary, it could be applied for adaptive radiotherapy and provides more accurate electron density for online dose calculation. Moreover, similar to the diagnostic fan beam CT, dual energy CBCT images can also provide more functional images, such as the effective atomic number images, monoenergetic images, and virtual unenhanced images. This will greatly enrich the application of CBCT, such as differentiating the tumor and normal tissue, catching the change of tumor composition, and improving the accuracy of patients' setup. It is significantly valuable in clinical application.

One possible limitation to the use of dual energy techniques is the potential for increased dose to the patient. The implementation of dual energy involves scanning the patient at a low energy and a high energy. Therefore the cumulative dose absorbed by the patient may be increased. However, the low mAs imaging parameters were used to minimize this increased dose in this study. For the high energy (120 kVp) and low energy (70 kVp), the exposure dose is about 0.4 mAs and 1.6 mAs per projection, so the increased dose is quite little compared with 1.6 mAs per projection for the clinical use of CBCT. Moreover, the valuable information gained by dual energy techniques is expected to be balanced against the slight increase in risk due to patient dose.

This method has six steps. For each patient, we need to scan twice with the high and low X-ray spectra (“Step 3”), so the scan time will increase by about 50 s. However, “Step 1” and “Step 2” can be performed only once for all patients and stored in the database for later improvement. Moreover, “Step 5” is the same with the routine CBCT procedure and the additional computation time for “Step 4” and “Step 6” is very short (about 12 s). So the total time increased is about 62 s compared with the routine CBCT procedure. Anyway, it is cost-efficient to improve electron density measurement of the CBCT. For clinical applications of our method, it is better to combine it in the CBCT software system to save time.

This study shows that, in the virtual phantom simulation with no noise, the method can obtain quite accurate values of electron densities. The errors are within 1%. However, in the phantoms experiments, the dual energy CBCT images are not so good. This is mainly because of the X-ray scatter which has not been corrected in the proposed method. If a scatter correction method could be combined with the proposed technology, its performance is expected to be better. The dual energy imaging quality improvement and its clinical applications warrant further investigation.

In conclusion, a novel dual energy CBCT imaging method to calculate the electron densities using high and low energy projection data was developed. It can acquire more accurate values and reduced the beam hardening artifacts obviously compared to the conventional CBCT. Therefore, it provides a platform potentially for dose calculation.

## Figures and Tables

**Table 1:** The results of the virtual phantom simulation.

Material	*ρ*_*e*_ (g·cm^−3^)		
	Calculated	Theoretical	Error (%)
Si	2.3071	2.3278	0.88
PMMA	1.2888	1.2945	0.44
PE	1.0559	1.0608	0.46
C	1.6839	1.6984	0.85
H_2_O	1.1091	1.1102	0.10
